# Hyperoxygenation as a Therapeutic Supplement for Treatment of Triple Negative Breast Cancer

**DOI:** 10.3389/fonc.2018.00527

**Published:** 2018-11-20

**Authors:** Jesse M. Mast, Periannan Kuppusamy

**Affiliations:** Department of Radiology and Medicine, Norris Cotton Cancer Center, Geisel School of Medicine, Dartmouth College, Lebanon, NH, United States

**Keywords:** breast cancer, tumor hypoxia, supplemental oxygen, EPR oximetry, paclitaxel, radiation

## Abstract

Triple-negative breast cancer (TNBC) refers to a group of biologically aggressive breast cancers that do not express estrogen, progesterone or epidermal growth factor receptor 2 hormone receptors. Each subset of TNBC has a unique molecular profile and may require specific treatments. A combination of surgery and chemotherapy followed by radiation therapy is the standard treatment mode for TNBC patients. Tumor oxygen status (hypoxia) is a key factor that may compromise the effectiveness of radiation treatment, as it is known that hypoxia can confer radiation resistance. In this study, we characterized MDA-MB-231 orthotropic xenograft tumors with respect to tumor oxygen level and their response to supplemental oxygen therapy in combination with paclitaxel and radiation therapy. We observed that the TNBC tumors became severely hypoxic (pO_2_ < 4 mmHg) within 1 week of tumor growth and responded poorly to administration of respiratory hyperoxygenation (100% O_2_) to mitigate hypoxia. However, periodic administration of supplemental oxygen (100% O_2_; 60 min/day for 21 days) showed a significant inhibitory effect on tumor volume when compared to control (1,023 ± 32 mm^3^ vs. 1,378 ± 114 mm^3^; *p* < 0.05). Combination of supplemental oxygen with paclitaxel and radiation therapy led to a significant reduction in tumor growth when compared to radiation alone (239 ± 40 mm^3^ vs. 390 ± 32 mm^3^; *p* < 0.05). The therapeutic enhancement by supplemental oxygen is possibly attributed to increase in tumor oxygenation with paclitaxel at the time of radiation treatment. These findings may have important implications in the understanding of the role of oxygen and supplemental oxygen therapy for the treatment of TNBC patients.

## Introduction

Breast cancer is the most common cancer among women worldwide, with an estimated 30% of new cancer cases in 2018 (1). Breast cancers are generally classified based on their hormone receptor status, namely, estrogen receptor (ER), progesterone receptor (PR), and human epidermal growth factor receptor 2 (HER2). There is a subtype of breast cancer that has none of these receptors, called triple-negative breast cancer (TNBC), which accounts for about 15% of all types of breast cancer. TNBC is a very aggressive cancer and is found very frequently in young women and women of African-American descent (2, 3). Hormone-positive breast cancers can be treated with a variety of drugs that exploit the reliance of these types of cancers on hormone signaling (4). Since TNBC does not have any of these receptors, hormone therapy is not an option for treatment (5, 6). Currently, the recommended regimen by the National Comprehensive Cancer Network for TNBC is a combination of surgery, radiatiotherapy and chemotherapy including a dose-dense doxorubicin+cyclophosphamide followed by Paclitaxel (Pax) weekly or every 2 weeks (7). Pax is a microtubule-stabilizing drug that is prescribed to treat a diverse range of cancers, is most commonly used for the treatment of TNBC (5).

Radiation therapy is a widely used modality for cancer treatment (8); however, it is non-specific and can cause damage to any cell, regardless of whether it is cancerous or healthy (9). The effect of radiation-induced cell killing can be amplified under certain conditions using radiation sensitizers (10–14). Molecular oxygen is an endogenous radiation sensitizer in tissues, including tumors, and hence tissue oxygen (pO_2_) is a critical factor in determining the efficacy of radiation (11, 14, 15). Locally advanced solid tumors are mostly low in oxygen (hypoxic), and the lack of oxygen has been implicated in causing radiation resistance and treatment failure (16–18). Tumor hypoxia is also known to promote aggressive phenotype, chemo-resistance, tumor growth and metastasis (19, 20). Accordingly, there has been several efforts to mitigate tumor hypoxia during radiation treatment (21, 22). Administration of hyperoxygen, usually by breathing or exposure to higher (>21%) oxygen at normobaric or hyperbaric conditions, has been studied in the clinic for over 4 decades to improve radiation efficacy (14, 23); however, neither a standard protocol for optimal efficacy nor adoption of it in clinical practice has been implemented as a standard care in the radiation oncology clinics. A Phase III clinical trial (24), named accelerated radiotherapy with carbogen and nicotinamide (ARCON) for the treatment of laryngeal cancer, reported some beneficial effect of hyperoxygenation for severely hypoxic tumors and concluded tumor oxygen is an important parameter to be measured for individual patients for the selection of effective treatment options.

Our laboratory and others have explored hyperoxygen treatment, by itself or as a potential option for enhancing other cancer therapies (21, 22, 25). We have shown that daily administration of hyperoxygen (100% oxygen; 2 atmospheres absolute, ATA; 90-min duration) daily for 21 days significantly inhibited the growth of ovarian cancer xenograft tumors, as much as cisplatin (21). The anti-tumor effect of hyperoxygenation was attributed to inhibition of STAT3 activation, which is a key step in the progression of ovarian cancer. We have also observed that hyperoxygenation, in combination with weekly administration of cisplatin, also significantly inhibited tumor growth; however, this group of mice had drastically reduced body weight when compared to other groups (21). In another study using breast cancer xenograft tumors with MCF-7 cells, we observed that repeated oxygenation (50% oxygen; 1 ATA; 180-min duration) daily for 20 days resulted in a significant regression of tumor growth without any adverse effect (25). The inhibitory effect of hyperoxygenation was attributed to oxygen-mediated rescue of conformationally mutant p53 under hypoxia to active p53 followed by induction of apoptosis. In both the cases the xenograft tumors were characterized with severe hypoxia, ~2 mmHg (19, 25), and mitigation of tumor hypoxia appears to have a beneficial therapeutic effect. Recently, Hatfield et al. reported that respiratory hyperoxia prevented the formation of lung metastases and improved long-term survival in a model of metastatic melanoma (22). They further showed that respiratory hyperoxia prevented the formation of melanoma lung metastases due to an activation of immune pathways, suggesting supplemental oxygen as an immunological co-adjuvant to combine with existing immunotherapies for cancer (22, 26, 27).

Despite several reports (28–31) that implicate hypoxia as a hallmark of TNBC, it is not known whether hypoxia is responsible for the aggressive growth and treatment failure of TNBC tumors. Therefore, the goal of the present study was to determine the oxygen status of TNBC as a function of tumor growth and establish its antitumor efficacy by itself or in association with standard therapies. We used MDA-MB-231 human TNBC xenograft tumors and measured tumor oxygen using EPR oximetry with OxyChip and evaluated the antitumor effect of hyperoxygenation in association with paclitaxel and radiation therapy. Our results showed that the TNBC xenograft tumor was severely hypoxic, and hyperoxygenation enhanced the therapeutic efficacy of paclitaxel and radiation. These findings highlight the significance of supplemental oxygen for the treatment of TNBC.

## Materials and methods

### TNBC xenograft tumors

The study used a total of 86 NOD-SCID-gamma (NSG) mice bred at Dartmouth Animal Resource Center. All animal experiments were performed with the approval of the Institutional Animal Care and Use Committee of Dartmouth College and conformed to the Guide for the Care and Use of Laboratory Animals, published by the National Institutes of Health (NIH Publication No. 86–23, Revised 1996). Mice were injected orthotopically with 3 × 10^6^ MDA-MB-231 cells. When the tumors were palpable, in about 27 days post-injection, the mice were separated into groups in such a way that the group-mean sizes of the tumors were as similar as possible. Tumor size was measured in two dimensions (width and length) with an electronic caliper and volume was determined using the formula, volume = (π/6) × width^2^ × length.

### Hyperoxygenation treatment

For hyperoxygenation treatment, tumor-bearing mice were placed in a custom-made air-tight chamber flushed with 100% oxygen gas and exposed to hyperoxygen for 1 or 2 h as desired. The control groups were kept in the room outside the chamber and received ambient oxygen (~21%) for the same duration.

### EPR oximetry using oxychip

OxyChip was prepared in the form a cylindrical pellet by embedding microcrystals of LiNc-BuO in polydimethylsiloxane (PDMS) and curing overnight at 70°C (32). OxyChips with approximate dimensions of 0.3-mm diameter × 1 mm length were calibrated and sterilized before implanting in tumors. OxyChips were implanted in the tumor at a depth of about 3 mm using a 23G syringe needle when the tumor volume was about 100 mm^3^. EPR measurements were taken at least 2 days after the placement of the chip. Tumor pO_2_ values were measured using an L-band (1.2 GHz) spectrometer equipped with a surface-loop resonator. Mice were kept under 2% isoflurane anesthesia and placed on a plastic bed plate such that the location of chip-implantation in the tumor was just beneath the loop, and approximately centered at the active surface of the loop resonator. Body temperature, during measurements, was monitored using a thermistor rectal probe, and maintained at 37 ± 0.5°C using a thermostatically controlled heated pad and a flow of warm air. EPR spectra were typically acquired continuously over a 20-min period each during baseline (30% oxygen breathing), hyperoxygenation, or back to baseline. All baseline measurements used 30% oxygen inhalation via nose-cone breathing. The higher concentration of oxygen was required to compensate for the drop in systemic oxygenation due to anesthesia. The EPR settings for these experiments were: microwave frequency, ~1.2 GHz; incident microwave power, 10–20 mW; scan time, 10 s; scan range, 0.5–10 G; modulation frequency, 27 kHz; modulation amplitude less than one-third of EPR line width.

### Paclitaxel and radiation treatment

Paclitaxel (TEVA brand) was sourced from the Dartmouth College Dick's House Pharmacy in lots of 30 mg (6 mg/ml) ready for injection. Ten milligram per kilogram Paclitaxel was injected intraperitoneally once per week. Radiation (6 Gy; Cs irradiator) was delivered to mouse kept under 2% isoflurane anesthesia.

### Statistical analysis

An unpaired *t*-test was used to compare the pO_2_ values at the different time points within the same group. The comparison reduces the effects of animal-to-animal heterogeneity and eliminates differences in the baseline pO_2_. The comparisons pO_2_ and tumor volumes were made using a student's *T*-test for unpaired samples. All data were expressed as Mean ± SEM. A *p* < 0.05 was considered statistically significant.

## Results

**Figure 1:**
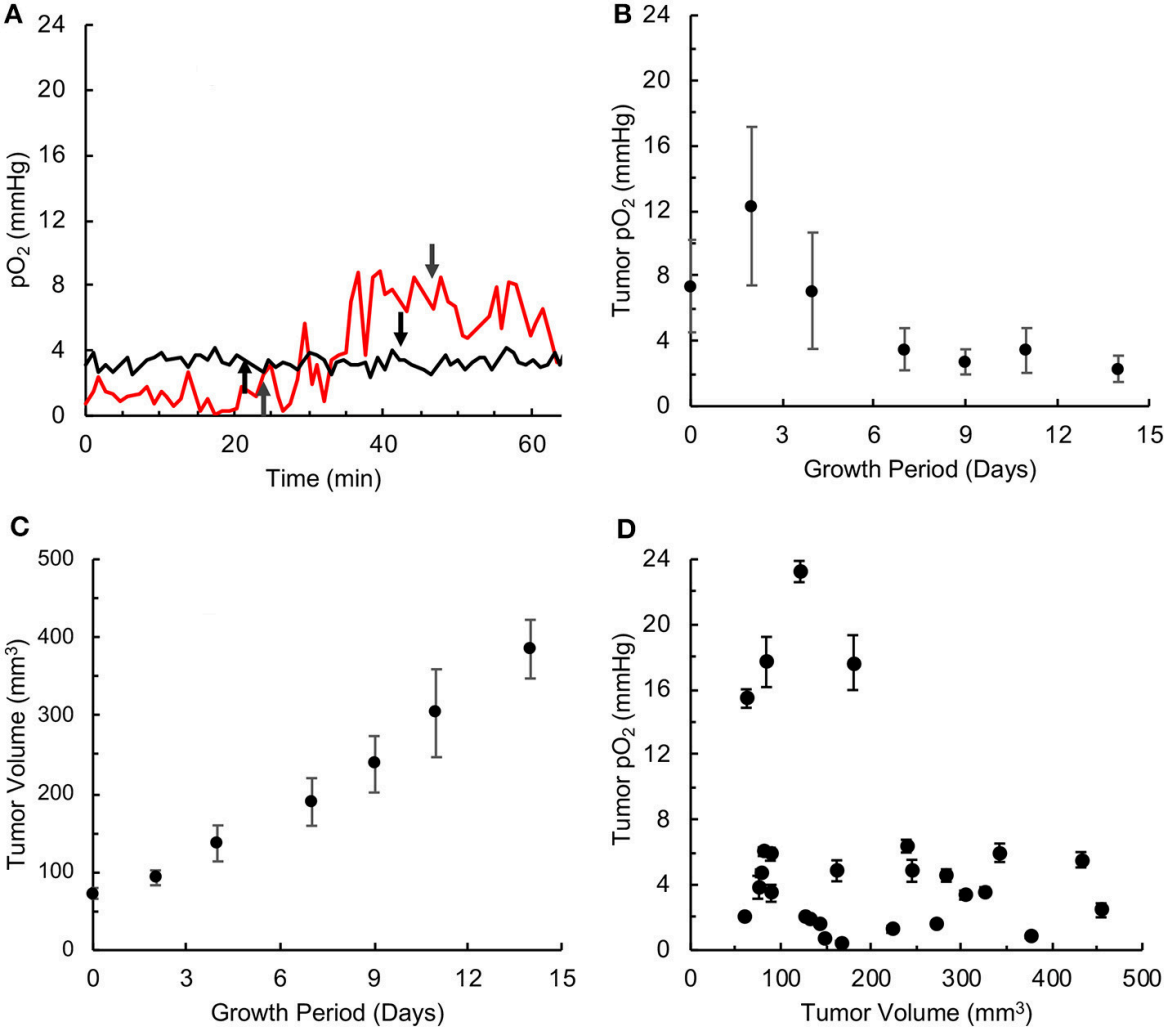
TNBC xenograft tumor is associated with severe hypoxia. Oxygen concentration (pO_2_) was measured by *in vivo* EPR oximetry in triple-negative breast cancer (TNBC) xenograft tumors, developed in mice using MDA-MB-231 cells. **(A)** Representative tracings of tumor pO_2_ values in mice breathing room air and 100% oxygen. In both cases, the mice were kept in room air for about 20 min, the breathing gas was switched to 100% oxygen at the time point indicated by the up-arrow and returned to room-air breathing 20 min later (indicated by down-arrow). In one case (red tracing), the tumor pO_2_ increased from about ~2 mmHg to ~8 mmHg at the end of 20 min of 100% oxygen administration, while in the other case (black tracing) the tumor oxygen was at ~4 mmHg and was non-responsive to hyperoxygen breathing. **(B)** Tumor pO_2_ values (Mean ± SEM, *N* = 4) measured over growth period. **(C)** Tumor volume (Mean ± SEM, *N* = 27) measured over growth period. **(D)** Relationship between tumor pO_2_ (Mean ± SEM of measurement) and volume for individual tumors. There is no correlation between tumor pO_2_ and volume (r = 0.269).

MDA-MB-231 TNBC xenograft tumors were characterized in terms of their size (volume), oxygen level (pO_2_) as a function of growth period, and their response to hyperoxygen treatment. Tumor pO_2_ values were measured using EPR oximetry with OxyChip implanted in the tumor. Typical measurements of tumor pO_2_ during room-air and hyperoxygen (100%) breathing conditions are shown in Figure 1A. Tumor pO_2_ decreased over time from an average of above 10 mmHg during the first few days down to below 4 mmHg rest of the period (Figure 1B). The spread in the pO_2_ values (deviation from the mean) also decreased as a function of tumor growth, followed for up to <400 mm^3^ of tumor volume in 14 days (Figure 1C). The pO_2_ readings and tumor volumes collected on the same day were compared to elucidate a putative relationship between tumor pO_2_ and tumor volume. While 4 of 16 pO_2_ readings were higher than the others before those tumors reached 200 mm^3^ in volume, there was no correlation between tumor volume and pO_2_. Overall, the results indicated that the TNBC xenograft tumors were severely hypoxic within a few days of growth

**Figure 2:**
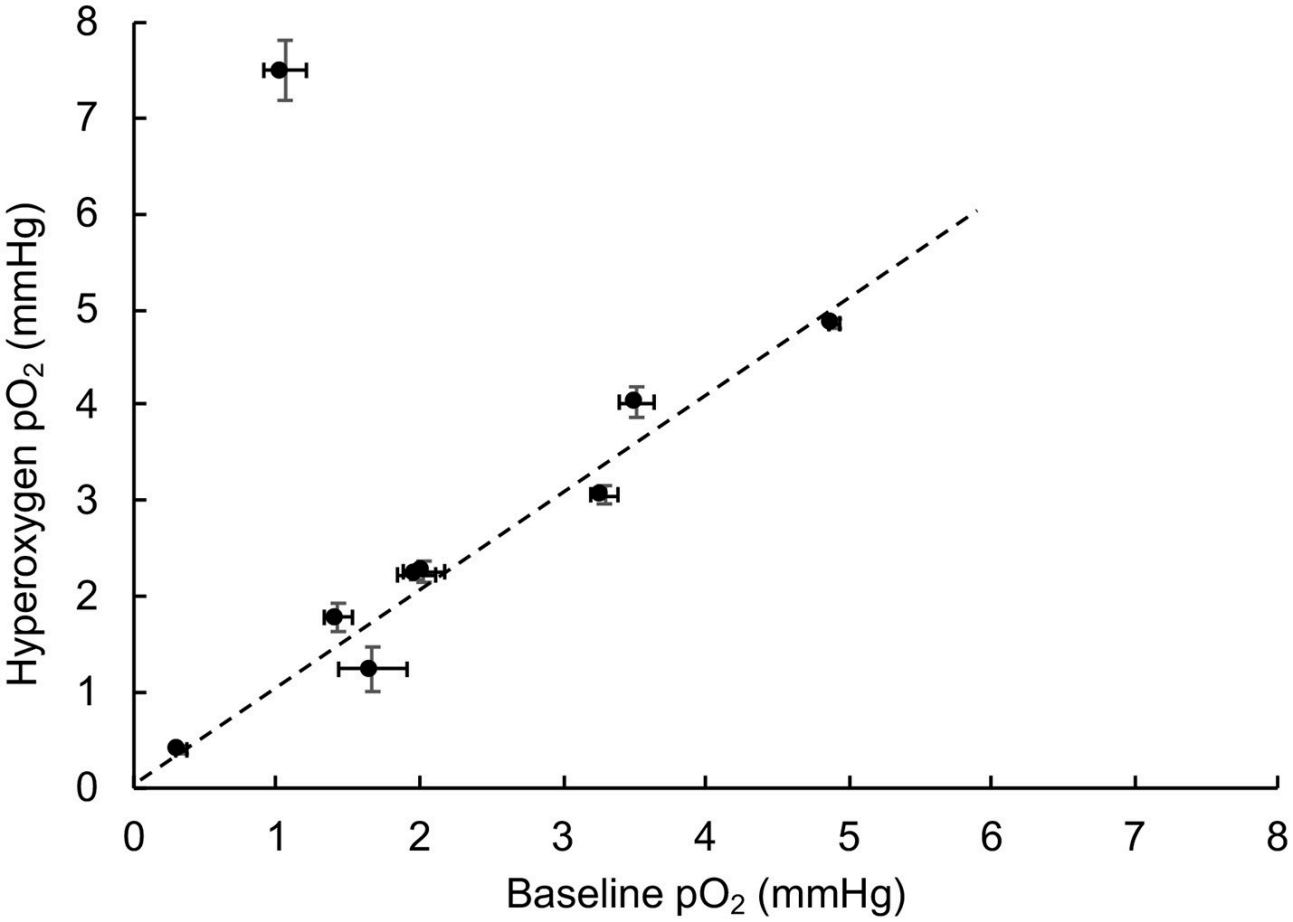
TNBC xenograft tumor does not respond to hyperoxygenation. Oxygen concentration (pO_2_) in TNBC xenograft tumors was measured during room-air breathing (baseline) and the end of 20 min of hyperoxygenation (100% oxygen) administered via nose-cone breathing. The plot shows the value of hyperoxygen pO_2_ as a function of baseline pO_2_. The error bars indicate SEM of measurement in the respective values. The results indicate that all, but one, of the tumors studied (*N* = 9) do not respond to hyperoxygenation suggesting that the TNBC tumors are not only severely hypoxic, but also resistant to hyperoxic intervention.

Tumor hypoxia is associated with aggressive growth and resistance to radiation and certain chemotherapies (19, 26, 33). Hence, mitigation of tumor hypoxia by hyperoxygen intervention is considered useful for improving treatment efficacy (14, 17, 21, 25, 34). To determine whether the TNBC tumor xenografts respond to hyperoxygenation, we used inhalation of 100% oxygen via nose-cone during pO_2_ measurements. After measuring baseline pO_2_ under room air conditions, the breathing gas was switched to 100% O_2_ and maintained for 20 min before switching back to room air. Figure 2 shows the maximum pO_2_ reached during hyperoxygenation as a function of baseline pO_2_. The results showed that all but one of the tumors did not show any significant change from the baseline pO_2_, indicating that these tumors do not respond to hyperoxygen intervention.

**Figure 3:**
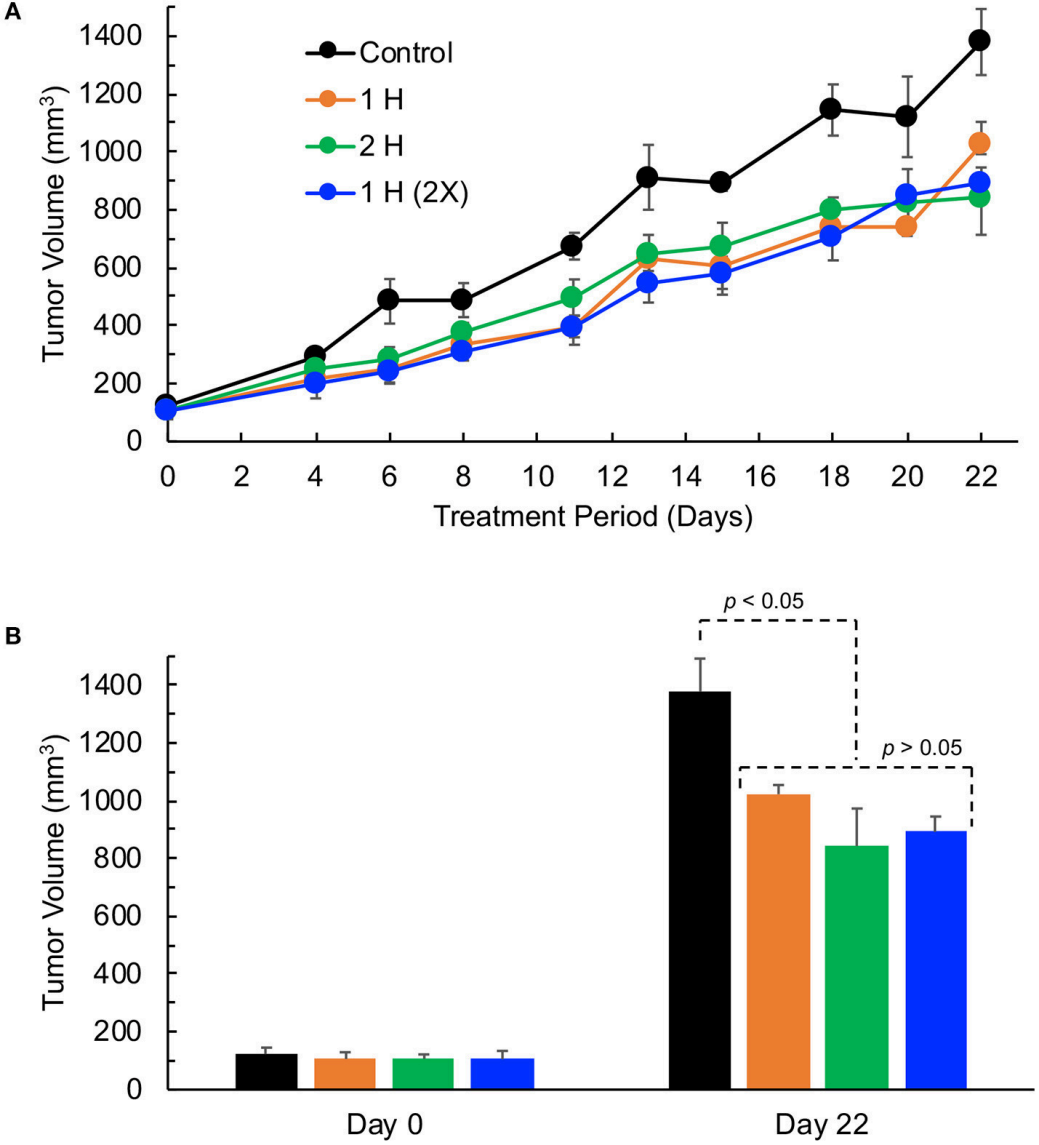
TNBC xenograft tumor growth is inhibited by hyperoxygen treatment. Mice bearing TNBC xenograft tumors were exposed to varying levels of hyperoxygen by periodic administration of 100% oxygen administered via nose-cone breathing for (i) 1 h per day (1 H), (ii) 2 h per day (2 H), and (iii) 1 h twice per day (2X). Data represent tumor volume (Mean ± SEM; *N* = 4–5) per treatment group for the entire period **(A)** and on days 0 and 22 **(B)**. All 3 modes of hyperoxygen administration show significant inhibition of tumor growth on day 22 (*p-*values: Control vs. 1H, 0.020; Control vs. 2H, 0.017; Control vs. 2X, 0.006); however, there are no significant differences in the tumor volumes among the treatment groups (*p*-values: 1H vs. 2H, 0.216; 1H vs. 2X, 0.067; 2H vs. 2X, 0.732) suggesting that 1 h per day (1 H) is as effective as the longer-duration (2 H) or multiple (2 X) treatments.

Supplemental oxygen treatment has been shown to be beneficial for effective cancer treatment (21, 22, 25). To determine the effect of supplemental oxygen on the growth inhibition of TNBC xenografts tumor, we treated tumor-bearing mice when the tumors were palpable (about 100 mm^3^ in size), with 1 h of 100% oxygen once per day (1 H), 1 h of 100% oxygen twice a day (2 X), or 2 h of 100% oxygen once per day (2 H). The results showed that treatment with 100% oxygen with all three cycling protocols significantly inhibited tumor growth at the end of 3 weeks of treatment (Figure 3). However, there were no significant differences among the treatment groups. In general, the supplemental oxygenation showed an inhibitory effect on tumor growth.

**Figure 4:**
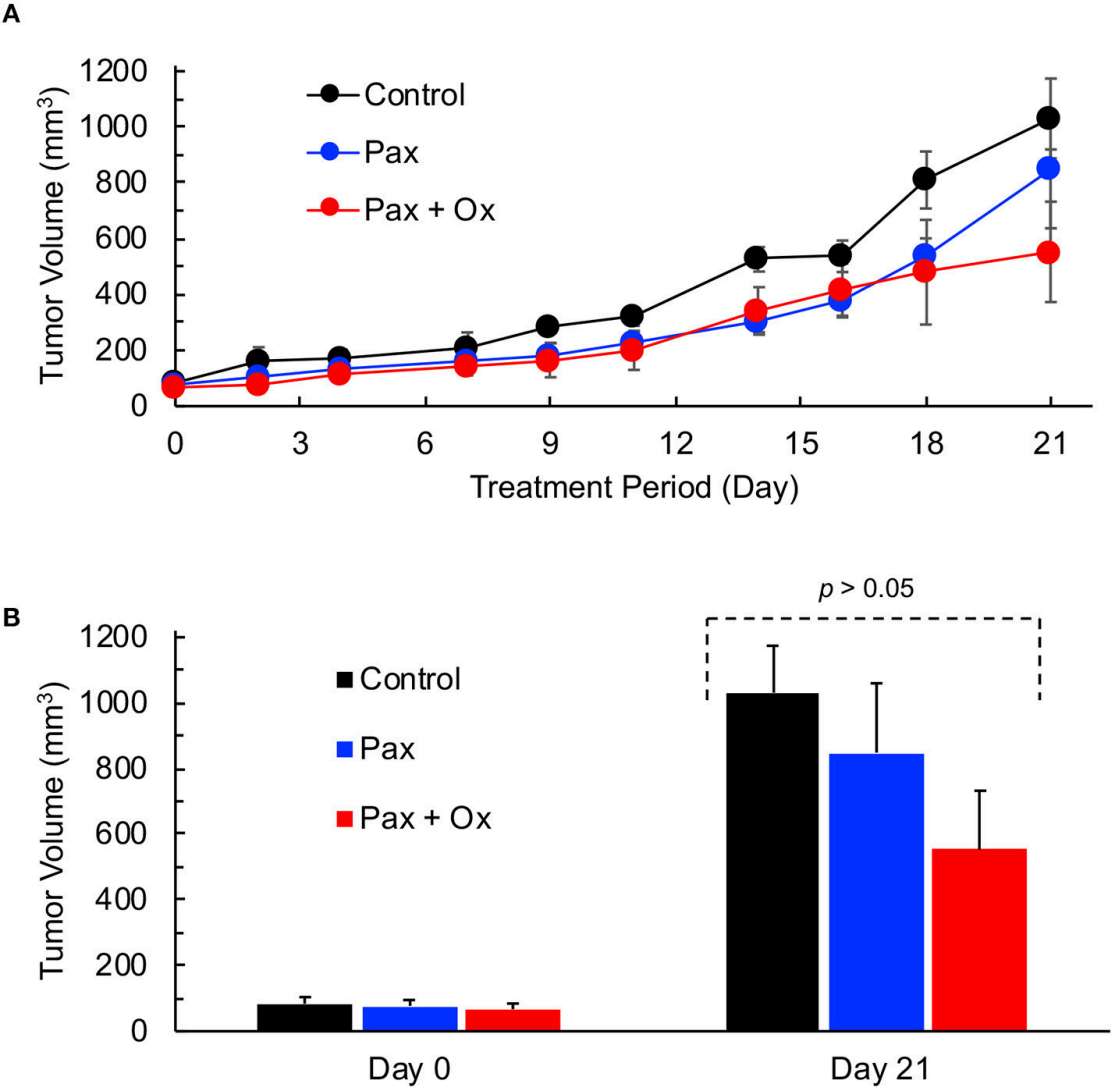
TNBC xenograft tumor growth is inhibited by hyperoxygenation and paclitaxel. Mice bearing TNBC xenograft tumors were treated with periodic hyperoxygenation, 1 h per day (Ox), and/or paclitaxel (Pax) as described in Methods. Data represent tumor volume (Mean ± SEM; *N* = 3–5) per treatment group for the entire period **(A)** and on days 0 and 21 **(B)** for the Control (no treatment), Pax and Pax + Ox. Although there seems to be an inhibition of tumor growth in the Pax and Pax + Ox group when compared to Control, the differences are not statistically significant (*p* values: Control vs. Pax, 0.513; Control vs. Pax+Ox, 0.106; Pax vs. Pax+Ox, 0.346).

**Figure 5:**
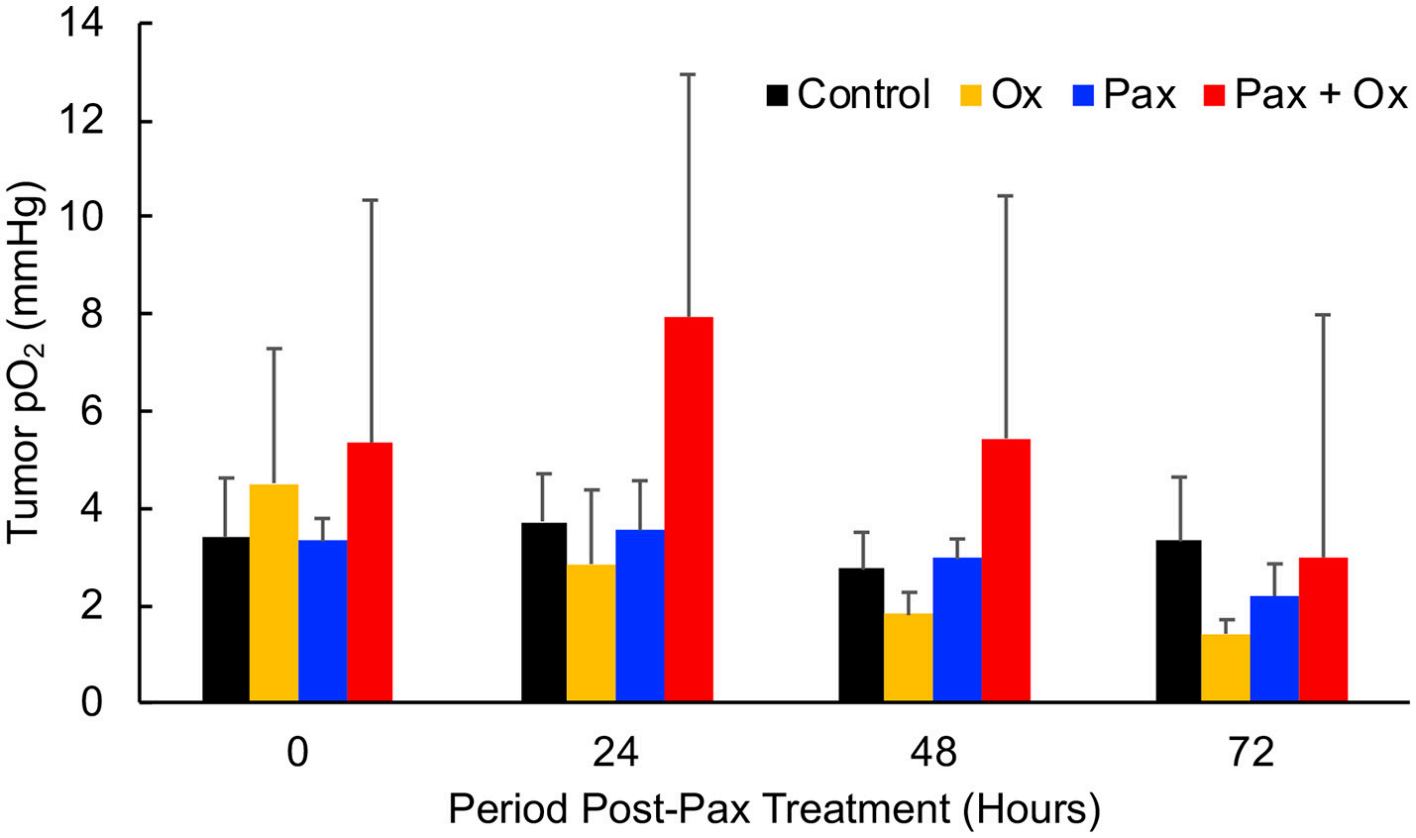
TNBC xenograft tumor pO_2_ responds to paclitaxel + hyperoxygen treatment. Mice bearing TNBC xenograft tumors were treated with periodic hyperoxygenation, 1 h per day (Ox), and/or paclitaxel (Pax) as described in Methods. Data represent mean pO_2_ values (Mean ± SEM; *N* = 4) measured at 0, 24, 48, and 72 h after treatment with paclitaxel (Pax), hyperoxygen (Ox), both (Pax + Ox), or no treatment (Control). There is no significant change (*p* > 0.05) in tumor pO_2_ in any group, but the Pax + Ox group does trend to have a higher pO_2_ 24 h after treatment.

Paclitaxel (Pax) is a preferred mode of drug used for the treatment for TNBC (5). We next determined the effect of Pax in combination with supplemental oxygen therapy. After 21 days of treatment, it was observed that both paclitaxel alone and paclitaxel with hyperoxygenation showed smaller tumors compared to untreated control; however, the differences were not significant (Figure 4). There was also no difference between Pax alone and paclitaxel plus oxygen treatments. It has been reported that administration of Pax to ER, PR, or HER2^+^ breast cancer patients led to increase in tumor oxygen after 9 cycles of weekly Pax (35). To determine whether Pax treatment can induce a similar effect in the TNBC xenograft tumor, we measured tumor pO_2_ for 3 consecutive days following Pax and Pax+oxygen treatment. Mice treated with once weekly paclitaxel, mice treated with daily hyperoxygenation therapy, and those treated with both paclitaxel and hyperoxygenation therapy were compared. It was observed that combination of paclitaxel and hyperoxygenation therapy led to a non-significant increase in tumor pO_2_ 24 h after injection of paclitaxel (Figure 5). Taken together, the Pax study revealed a modest inhibition of tumor growth and increase in tumor pO_2_ within 24–48 h of Pax+oxygen treatment.

**Figure 6:**
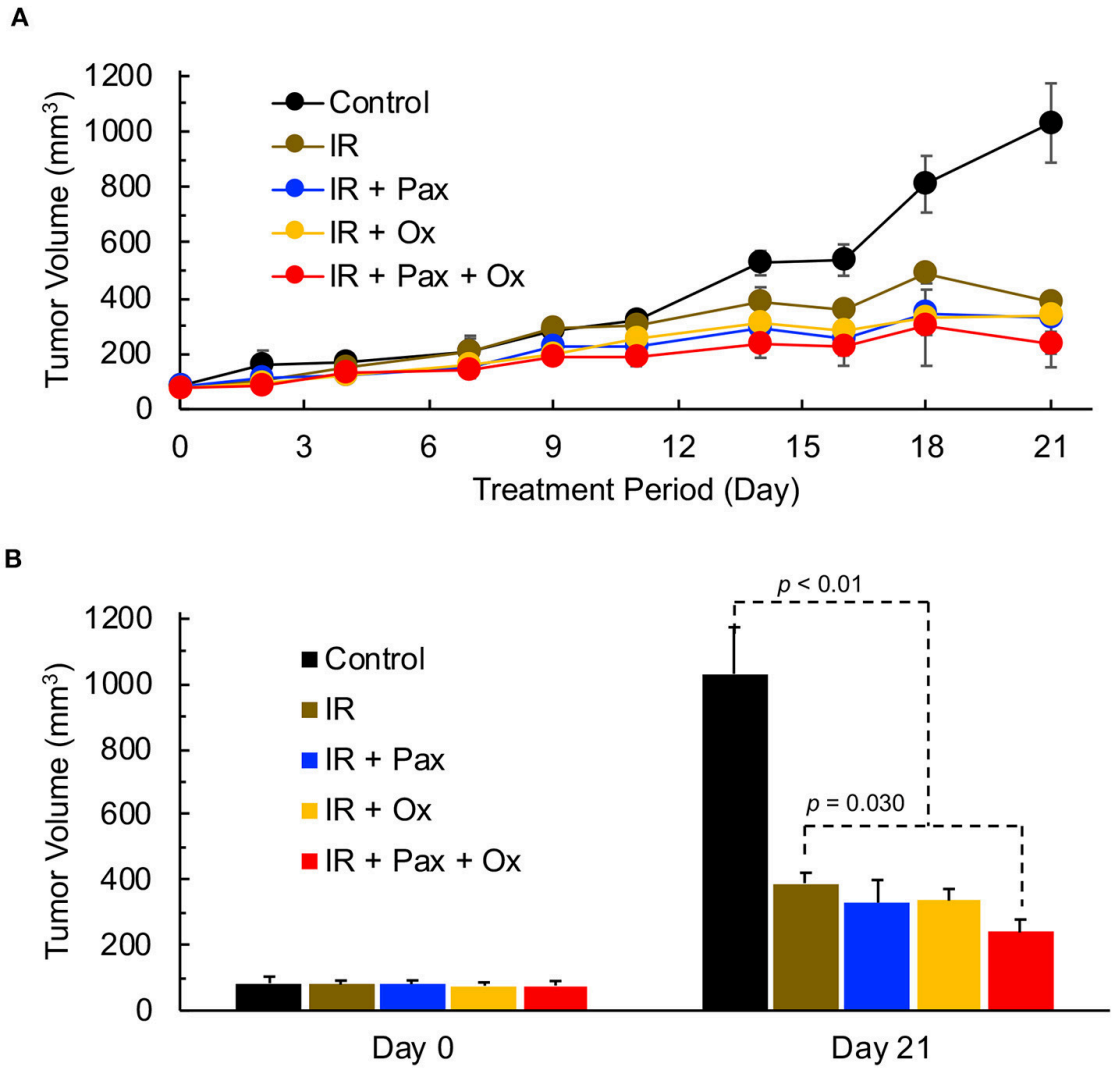
TNBC xenograft tumor growth is effectively inhibited by a combination of radiation, paclitaxel and hyperoxygenation treatments. Mice bearing TNBC xenograft tumors were treated with periodic hyperoxygenation, 1 h per day (Ox), and/or paclitaxel (Pax) in addition to radiation (IR) treatment once per week with 6 Gy. Data represent tumor volume (Mean ± SEM; *N* = 3–6) per treatment group for the entire period **(A)** and on days 0 and 21 **(B)** for the Control (no treatment), IR, IR+Pax, IR+Ox, and IR+Pax+Ox groups. All four treatment groups show significantly smaller volume than control group (*p*-values: Control vs. IR, 0.012; Control vs. IR+Pax, 0.012; Control vs. IR+Ox, 0.003; Control vs. IR+Pax+Ox,0.0004). Tumor group treated with IR+Pax+Ox shows significantly smaller tumor volume than that treated with radiation alone (*p* = 0.030).

We next determined whether the increase in tumor pO_2_ upon treatment with a combination of Pax and oxygen would enhance the efficacy of radiation treatment. The addition of paclitaxel and/or hyperoxygenation therapy to local irradiation of TNBC tumors was performed to determine efficacy of the combination treatment. Mice were treated with 6 Gy of local irradiation 48 h after injection of paclitaxel. All combinations of irradiation, hyperoxygenation therapy, and paclitaxel led to a significant decrease in tumor size 21 days after treatment as compared to control (Figure 6). In addition, the Pax+oxygen treatment led to significantly lower tumor volumes compared to radiation alone, suggesting that paclitaxel in combination with hyperoxygenation may provide an effective sensitizer for radiation therapy.

## Discussion

A salient discovery of our study is that the TNBC xenograft tumors are severely hypoxic, and further that hypoxia seems to occur at very early stages of tumor development. The tumors respond very poorly to hypoxia mitigation by hyperoxygenation. Combination of paclitaxel and hyperoxygenation treatments showed a modest increase on tumor pO_2_ and improvement of efficacy of radiation therapy. Our findings suggest that supplemental oxygen therapy along with paclitaxel can improve radiation efficacy and treatment outcomes.

The measurement of very low levels of tumor pO_2_ is made possible by the use of EPR oximetry with OxyChip. The method provides accurate, reliable, and repeated measurements of absolute value of oxygen concentration in tissues. The sensitivity of the method is particularly high at low pO_2_ levels. The pO_2_ levels we observed in the TNBC xenograft tumors were very low (<4 mmHg) indicative of severe hypoxia. These values were comparable to other xenograft tumors including ovarian cancer (A2780; ~2 mmHg), head and neck cancer (FaDu; ~4 mmHg), and breast cancer (MCF-7; 1.8 mmHg) that we have measured using EPR oximetry (19, 25, 36). The data from TNBC tumor also showed that tumor pO_2_ falls off rapidly with tumor growth; for example, it becomes <4 mmHg when the tumor size is 200 mm^3^ and plateaus thereafter. Hence, we did not continue to measure pO_2_ beyond 14 days of growth or for tumor volumes >400 mm^3^.

Another notable finding of our study is the lack of tumor response (pO_2_) to transient hyperoxygenation using 100% oxygen breathing. Only 1 out of 9 tumors measured showed an increase in tumor pO_2_ during hyperoxygenation. This lack of response to hyperoxygenation may be due to a variety of reasons, including poor or dysfunctional tumor vasculature or interstitial fluid pressure (35). Mitigation of tumor hypoxia during radiation therapy is considered as a viable strategy to sensitize hypoxic tumors to radiation (14, 33, 34); however, the poor response shown by the TNBC xenograft tumors seems to suggest that these tumors cannot be sensitized to radiation by transiently increasing their oxygenation.

On the other hand, the TNBC xenograft tumors appear to be responding to supplemental oxygen therapy by daily administration of 100% O_2_ for an hour or two. Oxygen treatment alone showed significant inhibition of tumor growth (Figure 3). Periodic or continuous exposure of mice to hyperoxygen has been shown to inhibit tumor growth and metastasis in a number of model tumors (21, 22, 25). Our group has shown growth inhibition of human ovarian cancer xenograft tumors in mice by periodic hyperbaric oxygen therapy (21). Mice exposed to hyperbaric oxygen (100% oxygen; 2 ATA; 90-min duration) daily, for up to 21 days showed a significant reduction in tumor volume, with no effect on body weight. We have also shown that repeated oxygenation at 30% O_2_ at normobaric conditions resulted in a significant regression of MCF-7 tumor xenografts without any adverse effect (25). In MCF-7 cells, re-oxygenation showed an eight-fold increase in cellular apoptosis. Hatfield et al. (22) demonstrated success using normobaric oxygen therapy to prevent the formation of melanoma lung metastases in an immune-competent model. Their model used 60% oxygen in a self-contained system that allowed the mice to be exposed to higher oxygen for the entire duration of their study. The treatment was highly effective, but their model was looking at metastasis formation, not primary tumor growth, and their mice were immunocompetent. They further showed that the effectiveness of their treatment was mainly due to immune mechanisms, a facet of tumor biology that our model is not capable of detecting.

Paclitaxel is frequently used as a chemotherapy for TNBC (5). Paclitaxel alone or in combination with supplemental oxygenation seem to have a moderate inhibitory effect on the growth of TNBC xenograft tumors. There is clinical evidence that paclitaxel may increase tumor pO_2_ by decreasing interstitial fluid pressure (35). Our results from TNBC xenograft tumors treated with paclitaxel did not show any evidence of increase in tumor oxygenation. On the other hand, paclitaxel combined with oxygen therapy showed a moderate, statistically insignificant, increase in tumor pO_2_ at 48 h after paclitaxel treatment. It is well-established that hypoxic tumors do not respond to radiation treatment as effectively as well-oxygenated tumors (16, 17, 18). Despite a non-significant increase in tumor pO_2_ with the addition of hyperoxygenation to paclitaxel, the addition of both led to a significant decrease in tumor growth when also treated with radiation therapy. Although the increase in tumor pO_2_ upon combination of hyperoxygen and paclitaxel treatment was not significant in our study, it appears to be biologically significant to enhance radiation efficacy. These findings indicate that the combination of oxygen, paclitaxel and radiation as an effective therapeutic regimen for TNBC. Although we have not investigated the possible mechanism underlying the phenomenon of cooperative effects of Pax, hyperoxygenation therapy, and irradiation therapy, it has been established in other models. The mechanisms involved in the context of TNBC, as well the possibility for synergism between the 3 explored therapies, have yet to be established, and will be explored in future works.

## Summary and conclusions

Our findings indicate that while TNBC is severely hypoxic, a combination of Pax and hyperoxygenation therapy can mitigate this hypoxia to a statistically non-significant level but have biologically significant effects when combined with radiation therapy. These findings could potentially be introduced into the clinic safely and quickly, with the goal of improving patient survival.

## Author contributions

JM, experiments, data analysis, interpretation and writing; PK, design, interpretation and writing.

### Conflict of interest statement

The authors declare that the research was conducted in the absence of any commercial or financial relationships that could be construed as a potential conflict of interest.

## Abbreviations

ATA: Atmospheres absolute
EPR: Electron paramagnetic resonance
ER: Estrogen receptor
HER2: Human epidermal growth factor receptor 2
IR: Ionizing radiation
NSG: NOD-SCID gamma
Pax: Paclitaxel
PDMS: Polydimethylsiloxane
pO_2_: Partial pressure of oxygen
PR: Progesterone receptor
TNBC: Triple negative breast cancer.
